# A Novel One-Step Fabricated, Droplet-Based Electrochemical Sensor for Facile Biochemical Assays

**DOI:** 10.3390/s16081231

**Published:** 2016-08-04

**Authors:** Yong Yao, Chunsun Zhang

**Affiliations:** MOE Key Laboratory of Laser Life Science & Institute of Laser Life Science, College of Biophotonics, South China Normal University, Guangzhou 510631, China; striveforhappiness@126.com

**Keywords:** one-step fabrication, droplet-based sensor, electrochemical sensing, biochemical assays

## Abstract

A simple, novel concept for the one-step fabrication of a low-cost, easy-to-use droplet-based electrochemical (EC) sensor is described, in which the EC reagents are contained in a droplet and the droplet assay is operated on a simple planar surface instead of in a complicated closed channel/chamber. In combination with an elegant carbon electrode configuration, screen-printed on a widely available polyethylene terephthalate (PET) substrate, the developed sensor exhibits a stable solution-restriction capacity and acceptable EC response, and thus can be used directly for the detection of different analytes (including ascorbic acid (AA), copper ions (Cu^2+^), 2′-deoxyguanosine 5′-triphosphate (dGTP) and ferulic acid (FA)), without any pretreatment. The obtained, acceptable linear ranges/detection limits for AA, Cu^2+^, dGTP and FA are 0.5–10/0.415 mM, (0.0157–0.1574 and 0.1574–1.5736)/0.011 mM, 0.01–0.1/0.008 mM and 0.0257–0.515/0.024 mM, respectively. Finally, the utility of the droplet-based EC sensor was demonstrated for the determination of AA in two commercial beverages, and of Cu^2+^ in two water samples, with reliable recovery and good stability. The applicability of the droplet-based sensor demonstrates that the proposed EC strategy is potentially a cost-effective solution for a series of biochemical sensing applications in public health, environmental monitoring, and the developing world.

## 1. Introduction

With the rapid development of portable lab-on-a-chip devices, miniaturized electrochemical (μ-EC) devices have attracted much attention due to their high detection sensitivity, insensitivity to light and color, ease of interface with the web, and ability to detect small volumes of sample [1]. Generally, early μ-EC devices were constructed on silicon, glass or polymer-based platforms, often requiring complicated chip-manufacturing techniques or multiple construction steps. Electrochemical microfluidic paper-based analytical devices (ePADs) were introduced by Dungchai et al. in 2009 as an alternative to first-generation μ-EC devices [2]. ePADs combine the advantages of paper-based devices (such as biodegradability, low cost, flexibility, light weight, reasonable mechanical strength, and pump-free sample transport) with those of EC systems.

To date, many approaches have been published to fabricate ePADs [3,4], and two different strategies have been used to integrate electrodes into paper-based devices: (i) the electrodes are fabricated directly on the patterned paper by silk-screen or other technologies (first-generation ePADs) [2,5,6,7,8,9,10,11,12,13,14,15]; (ii) the electrodes are fabricated on a separate substrate, and then this substrate is integrated with the patterned paper (second-generation ePADs) [16,17,18,19,20,21,22,23]. For the former, the fabrication of the electrodes must be compatible with that of the paper-based devices. Moreover, the electrode matrix occupies some of the volume that would otherwise be occupied by fluid. The latter strategy may open new possibilities for device configuration, and further improve detection limits. However, it involves a chip-bonding step, increasing the fabrication complexity and cost as well as the risk of interference in the biochemical assays. Moreover, the paper fiber blocks a portion of the electrode surface, resulting in a ~30% decline of the detection signal [16]. Regardless of the strategy, the cellulose matrix in ePADs largely blocks convection, acts as a barrier to diffusion, and occupies some of the volume that would otherwise be occupied by fluid [4]. A recent study also found that the pore size of the paper affected the ionic equilibria and formation of a potential at the electrode/sample interface [24]. Therefore, it is reasonable to question the necessity of using patterned paper in EC sensing.

Carbon ink-based electrode fabrication has been presented in two recent works [25,26], offering low cost and ease of operation. Differently from the EC sensors reported by Choi and co-workers [25,26], a novel droplet-based EC sensor has been developed in this work, and it is simpler, less expensive and less time-consuming than ePADs. The carbon electrodes of the sensor are one-step screen-printed onto a polyethylene terephthalate (PET) surface. Thanks to the electrode configuration and the hydrophobic/hydrophilic difference between the carbon electrode and the PET substrate, a droplet of EC solution can be precisely situated on the planar, screen-printed electrode (SPE)-containing surface, in contrast to the case of ePADs or early EC microdevices. Through characterization of their solution-restriction behaviors and cyclic voltammograms, the sensors displayed good reproducibility and efficiency. Detection of ascorbic acid (AA), copper ions (Cu^2+^), 2′-deoxyguanosine 5′-triphosphate (dGTP) and ferulic acid (FA) using different EC methods was achieved with excellent reproducibility, good linearity and acceptable sensitivity. Finally, the droplet-based EC detection of AA in commercial beverages and Cu^2+^ in water samples is demonstrated.

## 2. Experimental Section

### 2.1. Materials and Reagents

Carbon ink (model number CNB-7) was purchased from Bohui New Material Technology Co., Ltd. (Xuzhou, China). Whatman #1 chromatography paper (200 mm × 200 mm, pure cellulose paper) was purchased from GE Healthcare Worldwide (Shanghai, China) and used with further adjustment of size. Polypropylene paper was provided by Supreme Development Co., Ltd. (Shenzhen, China). A4 office paper was produced by Guangzhou Zhimeng Office Supplies Co., Ltd. (Guangzhou, China). Potassium chloride was obtained from Sigma-Aldrich (St. Louis, MO, USA). Ferrocene carboxylic acid (FCA) and AA were bought from J & K (Beijing, China). Sodium acetate trihydrate, phosphoric acid, acetic acid, boric acid, copper(II) sulfate pentahydrate, dGTP, FA and phosphate buffer solution (PBS, 20×) were purchased from Sangon Biotech Co., Ltd. (Shanghai, China). Sodium hydroxide was produced by Guangzhou Chemical Reagent Factory (Guangzhou, China). PET (0.1 mm thickness) was obtained from Hongmei Film Co., Ltd. (Shenzhen, China).

### 2.2. Apparatus

The screens were custom-made by the Lianchang Printing Equipment Shop (Guangzhou, China). An oven (DHG-9035A) was bought from Tensuclab Instruments Manufacturing Co., Ltd. (Shanghai, China). A water-purification system (ELGA PURELAB^®^ Option-R15, London, UK) was used to obtain deionized water. EC measurements were performed using a handheld CHI 1242B bipotentiostat (Shanghai CH Instruments, Shanghai, China).

### 2.3. Design and Preparation of the Sensor

**Figure 1:**
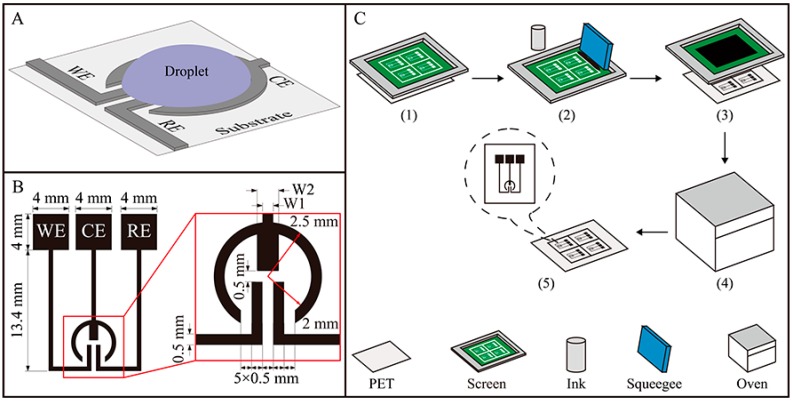
(**A**) Design principle of the droplet-based electrochemical (EC) sensor; (**B**) Three-electrode configuration and component sizes of the sensor; (**C**) Schematic illustration of the screen-printing process for fabrication of the droplet-based sensor: (1) the screen is placed on the substrate (polyethylene terephthalate (PET) or other); (2) carbon ink is dropped onto the screen, and then is rubbed with a squeegee; (3) the substrate is separated from the screen; (4) the screen-printed substrate is placed into the oven for baking; and (5) the desired sensor is formed for EC analysis.

In our design, a planar surface structure is crucial for the EC assay. Such a structure can be designed to possess several advantageous features: (1) it can strongly stabilize the droplet of EC solution on the specific reaction zone; (2) a three-electrode EC system, which is the most widely used EC format, can be provided; (3) the structure enclosing the droplet of the solution can be one-step fabricated, enabling the assay to be highly cost-effective. Figure 1A shows the design of the proposed droplet-based EC sensor.

Briefly, by making full use of the electrode configuration and the difference in hydrophilicity between the carbon electrode and the PET substrate, the droplet of EC solution is precisely situated on a circle defined by the counter electrode (CE), while the CE circle, working and reference electrodes (WE, RE) constitute a three-electrode system. In the present case (Figure 1B), the WE is designed to be as close as possible (about 0.5 mm) to the RE to minimize the effect of uncompensated resistance between the WE and RE. In addition, the clamp-shaped CE, which has a geometric area of 5.53 mm^2^, 6.41 mm^2^ or 7.28 mm^2^, is designed to be larger than the WE (geometric area 1.08 mm^2^) and RE (geometric area 1.08 mm^2^) to allow unlimited current transfer in the circuit. The CE has one straight handle, and two curved arms with or without convex bodies. To optimize the CE, the number (n) and width (W2) of the convex bodies as well as the width of the handle (W1) will be studied. The preparation of the sensor is straightforward. As shown in Figure 1C, carbon electrodes were one-step fabricated onto the PET or other substrates (Whatman #1 chromatography paper, office paper and polypropylene paper) by the simple screen-printing technology, and then their patterns were baked for <10 min in a 100 °C oven. The resulting sensors can be used without further modification.

### 2.4. Analytical Assays

Before the EC assays, a quantity of dye solution (3 or 6 μL) was added onto the developed sensor to evaluate its ability to effectively form a droplet on the desired structure. It should be noted that an appropriate volume of dye solution was used to form the reaction droplet, to ensure that the solution did not overflow from the CE of the sensor.

EC measurements of the four analytes were carried out with four different EC methods to evaluate the potential multifunctional application of the EC sensor, in which a 3 μL droplet of the corresponding solution was applied. Figure S1 shows a photograph of the measurement setup, which consists of a handheld potentiostat, screen-printed sensor and personal computer. The detailed analytical assays were as follows: square-wave voltammetry (SWV) with 5 Hz frequency, 25 mV amplitude, 4 mV potential increment and −0.4 to 1.2 V scanning potential was used to analyze AA (0–10 mM) in 100 mM acetate buffer solution (ABS) (pH 5.0). Square-wave anodic stripping voltammetry (SWASV) was performed for the detection of Cu^2+^ (0–1.5736 mM in 100 mM ABS (pH 4.5)). The experimental conditions were as follows: deposition potential −0.8 V, deposition time 120 s, equilibration time 10 s, amplitude 25 mV, potential increment 4 mV, frequency 5 Hz, and scanning potential −0.8 to 0.4 V. At open circuit, dGTP of 0–0.1 mM in 200 mM Britton–Robinson buffer solution (BRBS) (pH 6.0) was first accumulated for 180 s, then its EC response was measured by differential pulse voltammetry (DPV) using the following conditions: pulse amplitude 50 mV, pulse width 0.05 s, pulse period 0.5 s, potential increment 4 mV, potential range 0 to 1.5 V. Cyclic voltammetry (CV) with a scan rate of 0.1 V/s ranging from −0.4 to 0.6 V was used to determine FA (0–0.515 mM) in 100 mM ABS (pH 5.0).

## 3. Results and Discussion

### 3.1. Electrode Design and Droplet Formation Associated with the Proposed Sensor

**Figure 2:**
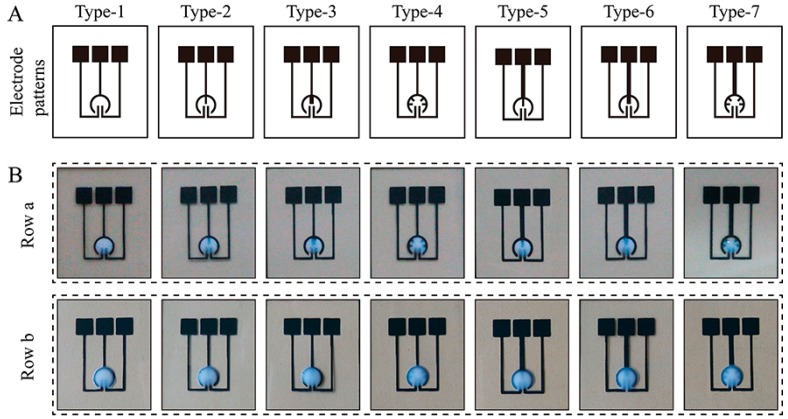
(**A**) Designs of electrode patterns with various types of counter electrode (CE); (**B**) Photograph of various droplet-based electrochemical (EC) sensors filled with 3 μL (**Row** **a**) or 6 μL (**Row** **b**) dye solution.

In this work, a simple yet effective solution-restriction strategy for the formation of a droplet has been organically coupled with an electrode configuration for EC sensing. Figure 2A shows the designs of seven electrode patterns. For each pattern, the liquid storage capacity of the sensor using a PET substrate was evaluated (Figure 2B). At 3-μL volume, the interior of the CE was filled with the dye solution (Row a). With the volume increasing to 6 μL, the solution covered the whole CE (Row b). In each case, all the electrode patterns achieved a stable, effective droplet formation, possibly because of the subtle design of the electrode structure and the choice of substrates. Specifically, the solution was blocked by the CE in the horizontal direction because the hydrophilicity of the CE material is greater than that of the PET substrate. Meanwhile, in the vertical direction, the waterproofing quality of the PET substrate prevented the permeation of the solution into it. In the following experiments, a solution volume of 3 μL was chosen to reduce the reagent consumption. The use of such small volumes for analysis was motivated by three further considerations: (1) taking into account the proposed solution-restriction mechanism, it is difficult for the sensor to carry out large-volume analysis; (2) the use of small volumes reduces the analytical cost; and (3) the present droplet-based analysis is intended to be compatible with other increasingly popular small-volume detection elements, such as droplet-based polymerase chain reaction (PCR) [27], in the future. As discussed later, the proposed EC sensor has been used for the determination of dGTP, which is usually an indispensable reaction substrate for PCR-based DNA amplification.

It is necessary to state that when Whatman #1 chromatography paper, office paper, or polypropylene paper was used as the substrate material, the formation of the droplet on the fabricated sensor was not successful. For chromatography paper and office paper, the solution spread quickly in the horizontal direction, while in the vertical direction the solution permeated into the paper substrate. For polypropylene paper, although the permeation of the solution was prevented in the vertical direction, its high hydrophilicity facilitated the solution diffusion in the horizontal direction. We also note that the formation of the sensor presented here did not require a separate processing step to construct the reaction chamber, in contrast to some other EC sensors (Table 1). In the products of the commercial supplier DropSens, for example, an insulating layer serves to delimit the EC cell and electrical contacts [28]. In general, the presented sensor does not cause a large difference in the contact area between the electrodes and the droplet. We found that a small perturbation (e.g., vibration) did not change the contact area substantially (Figure S2 and Supplementary Video).

### 3.2. Electrochemical Characteristics of the Proposed Sensor

**Figure 3:**
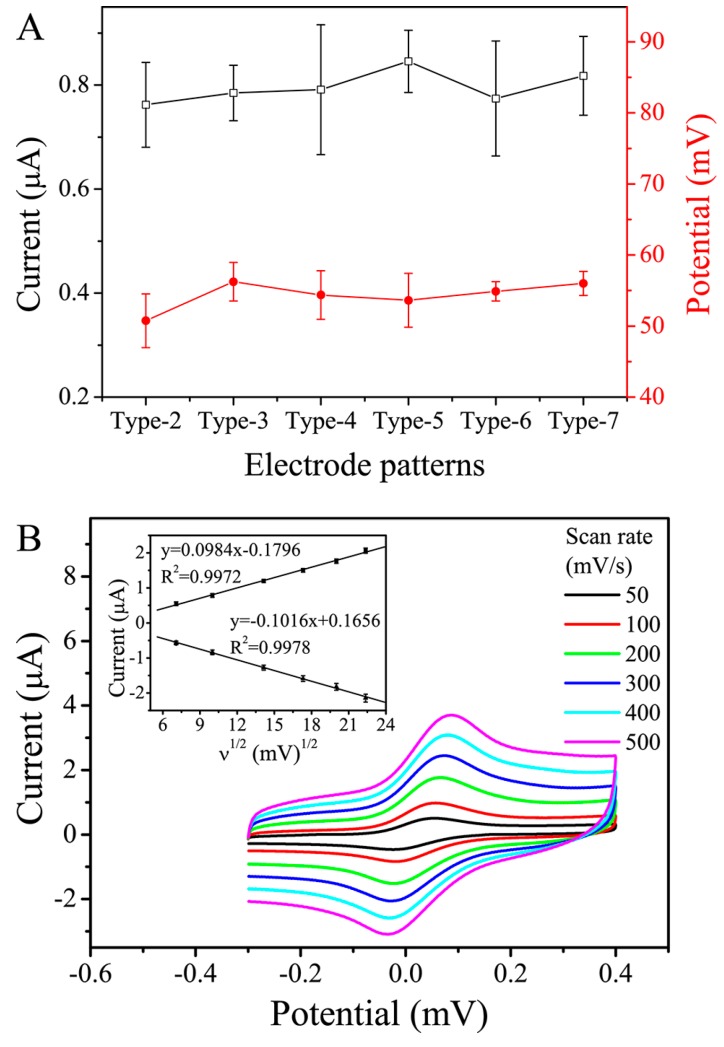
(**A**) Cyclic voltammetry (CV) peak current or peak potential on electrochemical (EC) sensors with various electrode patterns (scan rate: 100 mV/s; potential range: −0.3 to 0.4 V versus carbon pseudo-reference electrode; buffer solution: 2.0 mM ferrocene carboxylic acid (FCA) in 500 mM KCl aqueous solution); (**B**) Cyclic voltammograms of 2.0 mM FCA in 500 mM KCl aqueous solution using the Type-3 EC sensor at various scan rates. The relationship between the peak current and the square root of the scan rate is shown in the insert. The error bar represents the standard deviation from eight independent measurements.

As a commonly used EC method, CV was applied to evaluate the EC behavior of the sensor, whose printed material (with a sheet thickness of ~18 μm) has a sheet resistance of ~85 Ω/square. Similar to the previous reports [25,26], the sheet resistance of the SPEs was measured using a standard two-point ohmmeter. A rectangular line of 1 mm × 10 mm (10 squares) was screen-printed, and then its bulk resistance was determined using the ohmmeter and was divided by 10. In the CV measurements, 2 mM FCA in 500 mM KCl was used as a model electroactive compound. For the Type-1 sensor (the CE of which has no convex body), the CV behavior was sporadic and uncontrollable, possibly due to the erratic connection between the EC solution and the CE. For the other types (Type-2 to Type-7), however, the sensor achieved an acceptable, stable CV performance (Figure S3). Figure 3A shows the relationship between the peak current or peak potential values and the electrode patterns. It was found that Types-3, 4, 6 and 7, whose values of W2 × n (i.e., the total geometric area of the convex bodies of the CE) were the same by design and appreciably larger than those of the others, showed a smaller deviation in peak potential. This may be attributable to their larger working area causing the CE to have a reduced current density, thus making the CE less polarizable. For Types-4 and 6, however, their peak currents had a slightly larger deviation. Currently, this phenomenon is difficult to explain clearly. Based on these observations, the Type-3 electrode pattern was selected for the following experiments.

The voltammograms displayed in Figure 3B further characterize the CV behavior of the Type-3 electrode pattern. The voltammogram of FCA shows a cathodic peak and an anodic peak in its potential range at the carbon WE. With increasing scan rate, the separation between the cathodic and anodic peak potentials increased from 76 mV to 121 mV, which are larger than the theoretical value of 59 mV. However, the corresponding peak current ratios, which could be determined by using the equation proposed by Olmstead et al. [35], were close to 1 at each scan rate. In addition, the insert in Figure 3B shows that the cathodic and anodic peak currents were linearly proportional to the square root of the scan rate between 50 and 500 mV/s. The above results show that the EC reaction at the presented electrodes is a diffusion-controlled and reversible redox process [36]. These phenomena are similar to those in previous reports [12,37], where the separation between the peak potentials significantly increased with increasing scan rate. We propose three possible reasons for this behavior. Firstly, although the WE is designed to be as close as possible to the RE, the effect of uncompensated resistance has not been eliminated. Secondly, the carbon ink formulation for the fabrication of the SPEs may adversely affect the electron transfer reactivity and overall EC performance of the resulting sensor [38]. Finally, the surface of the uncoated SPEs in the present work may exhibit a certain hydrophobicity, which would not be beneficial to the uniform distribution of the aqueous FCA solution, and thus to the electrode response [37].

### 3.3. Biochemical Assay Applications of the Sensor

**Figure 4:**
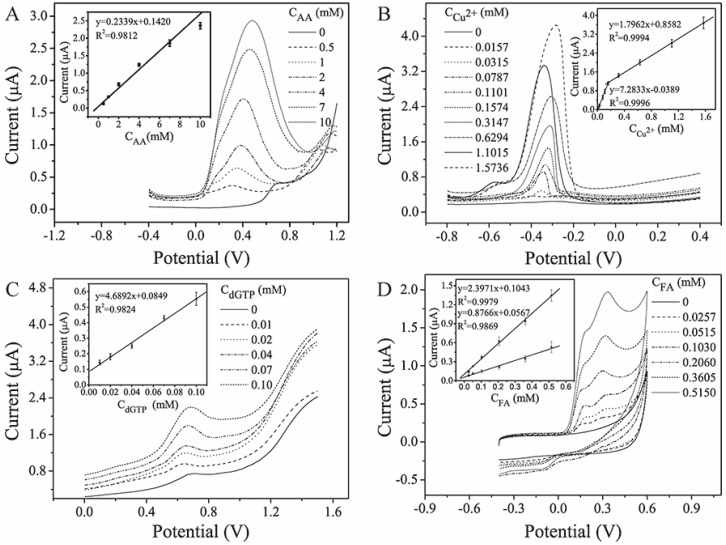
Comparison of electrochemical (EC) methods with the proposed sensor for determination of targets of interest: square-wave voltammetry (SWV) for determination of ascorbic acid (AA) (0–10 mM) in 100 mM acetate buffer solution (ABS) (pH 5.0) (**A**); square-wave anodic stripping voltammetry (SWASV) for determination of Cu^2+^ (0–1.5736 mM) in 100 mM ABS (pH 4.5) (**B**); differential pulse voltammetry (DPV) for determination of 2′-deoxyguanosine 5′-triphosphate (dGTP) (0–0.1 mM) in 200 mM Britton–Robinson buffer solution (BRBS) (pH 6.0) (**C**); and cyclic voltammetry (CV) for determination of ferulic acid (FA) (0–0.515 mM) in 100 mM ABS (pH 5.0) (**D**). The error bar represents the standard deviation from eight independent measurements.

As a common antioxidant, AA plays a significant role in metabolism in the human body, and affects many essential physiological processes, such as cell division and gene expression [39]. Therefore, a rapid and highly selective sensing method for the direct detection of AA is urgently needed, in which the oxidation of AA to dehydroascorbic acid would be the most likely pathway. Figure 4A shows the SWV response of the sensor with AA concentrations ranging from 0 mM to 10 mM. It was found that the oxidation peak currents increased with increasing AA concentrations, with a good linearity (*R*^2^ = 0.9812, *n* = 8) and an acceptable limit of detection (LOD) of 0.415 mM. In this work, the LOD was defined as the concentration producing a signal three times the standard deviation of a blank.

The determination of copper species is important in both environmental and process monitoring. The SWASV method was applied for Cu^2+^ analysis. The stripping voltammograms show well-defined peaks and strong signals over the two ranges of 0.0157–0.157 mM and 0.157–1.57 mM (Figure 4B), giving two excellent linear relationships (*R*^2^ (1) = 0.9996, *R*^2^ (2) = 0.9994, *n* = 8) and an LOD as low as 0.011 mM. Thus, the sensor has the potential to be used for monitoring the quality of drinking water (≤0.0157 mM, GB5749-2006, China).

dGTP is one of the most important substances for DNA synthesis and several cellular functions, but is rarely detectable by EC methods. In our case, the more sensitive DPV method was used to monitor the level of dGTP, with typical voltammograms shown in Figure 4C. A good linear relationship was established between the oxidation peak current and the dGTP concentration in the range of 0.01–0.1 mM (*R*^2^ = 0.9824, *n* = 8), and the LOD of the assay was calculated to be 0.008 mM. Generally, the concentration of dGTP used in the polymerase chain reaction (PCR) protocol, a widely used technique for nucleic acid amplification, is approximately 0.2 mM [40], and thus the proposed sensor has the potential to be used for monitoring the PCR process.

FA is an important phenolic antioxidant, and its content in food and pharmaceutical cosmetics must often be precisely and accurately evaluated. Here, a simple, effective CV protocol was employed to detect FA (Figure 4D). No redox peak was observed in the absence of FA. When FA at concentrations of 0.0257–0.515 mM was present, however, two anodic peaks were observed at 0.18 ± 0.02 V and 0.30 ± 0.03 V. Their respective corresponding peak currents increased with increasing FA concentration, showing a good linearity (*R*^2^ (1) = 0.9979, *R*^2^ (2) = 0.9869, *n* = 8) and an acceptable LOD (0.024 mM).

As seen from the above results, the proposed droplet-based EC sensor can be used to perform biochemical assays of several species using various EC protocols. On the whole, the obtained analytical performances, such as linear detection range and LOD, are comparable to or only slightly worse than those obtained by several other EC analytical methods (Table 2). However, the presented sensor can be one-step fabricated for facile biochemical assays, associated with a simple and cheap process and acceptable EC characteristics. It should be noted that unlike in most of the above-mentioned EC methods, the WE of the present droplet-based sensor does not undergo any modification, and thus its applications may be limited. Fortunately, various surface modification strategies, such as layer-by-layer assembly and self-assembled monolayers, could potentially be used for the decoration and modification of the sensor to further improve its detection performance.

### 3.4. Determination of AA or Cu^2+^ in Real Samples

To verify the applicability of the proposed droplet-based EC sensor, two commercial beverages were used as model samples for the detection of AA. Similarly, tap water and wastewater were used as two real water samples for the analysis of Cu^2+^ levels. To demonstrate that the presented sensor is a suitable alternative to existing methods, the samples tested by the proposed method were firstly verified in parallel using the standard methods. The detection results are shown in Table 3. Here, the respective experimental conditions were similar to those described above. In addition, the beverages were spiked with 1.5 or 2 mM AA, and the concentrations of Cu^2+^ spiked into wastewater and tap water were 0.05 mM and 0.016 mM, respectively. For all samples with no addition of analyte, the level of AA or Cu^2+^ measured by the proposed droplet-based EC sensor was in good agreement with that obtained by the corresponding standard method. For beverages A and B, the recoveries of AA were 105.5% and 107.3%, with an RSD of <6.5%. For detection of Cu^2+^, the recovery of Cu^2+^ in wastewater was 106.0%, with an RSD of 6.3%. However, the recovery of Cu^2+^ in tap water could not be achieved. Possible reasons for this are that the tap water sample did not contain copper ions, or that the sensor's sensitivity is too low. Therefore, it may be reasonable to conclude that the proposed sensors are more suitable for the detection of AA in commercial beverages and Cu^2+^ in wastewater.

## 4. Conclusions

We have developed an EC sensor by integrating a droplet-based platform with carbon electrodes for quantitative biochemical assays. The preparation of the sensor is extremely simple, as complex treatment procedures involving the use of multiple chemicals and modern equipment are avoided. The proposed sensor can detect various analytes of medical, environmental and food safety significance, making it highly advantageous because of the simplicity and cost-effectiveness of its preparation. The proposed one-step fabricated EC sensor has several obvious advantages: (1) there is no need to fabricate an EC chamber/channel specifically by using etching or other technologies; (2) professional equipment/training is not necessarily required; (3) PET, as the only required substrate material, is relatively cheap; (4) the volume consumption of the assay solution can be as low as 3 μL; (5) PET is superior in “wet strength”, in contrast to paper; and (6) the proposed sensors are highly promising for fabrication in batch mode. However, the proposed sensor has some drawbacks. For example, PET is not easily degraded; moreover, the sensor may present difficulties in sensing applications requiring device flexibility. In future, by making full use of the sensor’s advantages and further combining the developed sensors with other previously demonstrated microfluidic and detection functions, we foresee that this novel sensing platform might serve as a useful tool for field-deployable analysis or point-of-care applications.

## Figures and Tables

**Table 1:** Comparison between the proposed electrochemical (EC) sensor and other EC sensors.

Sources	Substrates	Materials for Construction of EC Cell	Pretreatment ^a^	Ref.
Dropsens	Alumina	Insulating layer	NA	[28]
Micrux	Glass	SU-8	NA	[29,30]
Self-made	Paper	PDMS	Yes	[31]
Self-made	Paper	SU-8	Yes	[2]
Self-made	Paper	Wax	NO	[32]
Self-made	PET	Hydrophobic ink	NO	[33]
Self-made	Polyester	UV curable dielectric and PDMS	NO	[34]
Self-made	PET	NO	NO	This work

^a^ Pretreatment indicates whether pretreatment is needed for the substrate before fabrication of an EC cell; NA: not available; PET: polyethylene terephthalate; PDMS: poly(dimethylsiloxane); SU-8: an epoxy resin.

**Table 2:** Comparison of detection performances between the proposed EC method and other analytical methods.

Analyte	EC Method	Linear Range (mM)	*R*^2^	LOD (mM)	Ref.
AA	DPV	0.5–2	0.9860	0.25	[41]
	CV	5–60	0.9940	5	[42]
	CV	0.4–6	0.9940	0.12	[43]
	SWV	0.5–10	0.9812	0.415	This work
Cu^2+^	Direct potentiometry	0.005–100	N/A	0.0047	[44]
	Direct potentiometry	0.05–100	N/A	0.05	[45]
	Direct potentiometry	0.01–100	N/A	0.008	[46]
	SWASV	0.0157–0.157	0.9996	0.011	This work
		0.157–1.57	0.9994		
dGTP	DPV	0.001–0.7	0.9980	0.001	[47]
	DPV	0.002–0.5	N/A	0.001	[48]
	DPV	0.004–0.24	N/A	0.002	[49]
	DPV	0.01–0.1	0.9824	0.008	This work
FA	DPV	0.0154–0.721	0.9994	0.005	[8]
	SWASV	0.005–1	0.9882	0.001	[50]
	CV	0.0257–0.515	0.9979	0.024	This work

EC-electrochemical; LOD-limit of detection; AA-ascorbic acid; DPV-differential pulse voltammetry; CV-cyclic voltammetry; SWV-square-wave voltammetry; N/A-not available; SWASV-square-wave anodic stripping voltammetry; and dGTP-2′-deoxyguanosine 5′-triphosphate.

**Table 3:** Determination of ascorbic acid (AA) or Cu^2+^ in real samples.

Analyte	Sample	Reference Method ^a^ Detected ^b^ (±S.D.) (mM)	Proposed Method				
			Detected (±S.D.) (mM)	Added ^c^ (mM)	Found ^d^ (mM)	RSD ^e^ (%)	Recovery ^f^ (%)
AA	Beverage A	2.051 (±0.114)	1.976 (±0.118)	2.0	4.086	6.4	105.5
	Beverage B	1.406 (±0.039)	1.319 (±0.117)	1.5	2.929	4.4	107.3
Cu^2+^	Waste water	0.040 (±0.002)	0.042 (±0.004)	0.05	0.095	6.3	106.0
	Tap water	<0.0002	Not detected	0.016	0.016	5.5	/

^a^ Reference method means that spectrophotometry and atomic absorption spectrometry were used for the detection of AA and Cu^2+^, respectively; ^b^ Detected is the amount of AA or Cu^2+^ in the unspiked real sample; ^c^ Added is the value that we added into the real sample; ^d^ Found is the value obtained in the spiked real sample; ^e^ RSD is the relative standard deviation calculated from eight independent experiments; ^f^ Recovery is the ratio of (Found − Detected)/Added.

## Supplementary Materials

The following are available online at http://www.mdpi.com/1424-8220/16/8/1231/s1: Figure S1: Photograph of the setup. The device principally consists of a personal computer (1), handheld bipotentiostat (2), and droplet-based electrochemical (EC) sensor (3); Figure S2: Photograph of the droplet-based electrochemical (EC) sensor before (A) and after (B) vibration; and Figure S3: Cyclic voltammograms of 2.0 mM ferrocene carboxylic acid (FCA) in 0.5 M KCl aqueous solution using Type-2 (A), Type-3 (B), Type-4 (C), Type-5 (D), Type-6 (E) and Type-7 (F) electrochemical (EC) sensors (scan rate: 100 mV/s; potential range: −0.3 to 0.4 V versus carbon pseudo-reference electrode). Here, eight independent measurements were performed for each pattern.
