# Supplementary material for: A Novel One-Step Fabricated, Droplet-Based Electrochemical Sensor for Facile Biochemical Assays
Source: Sensors (Basel). 2016 Aug 4;16(8):1231. doi: 10.3390/s16081231 (PMC5017396; doi:10.3390/s16081231)
Supplement: Supplementary file 1 [file sensors-16-01231-s001.zip › sensors-16-01231-supplementary.pdf]

Supplementary Materials: A Novel One-Step Fabricated, Droplet-Based Electrochemical Sensor for Facile Biochemical Assays

Yong Yao and Chunsun Zhang

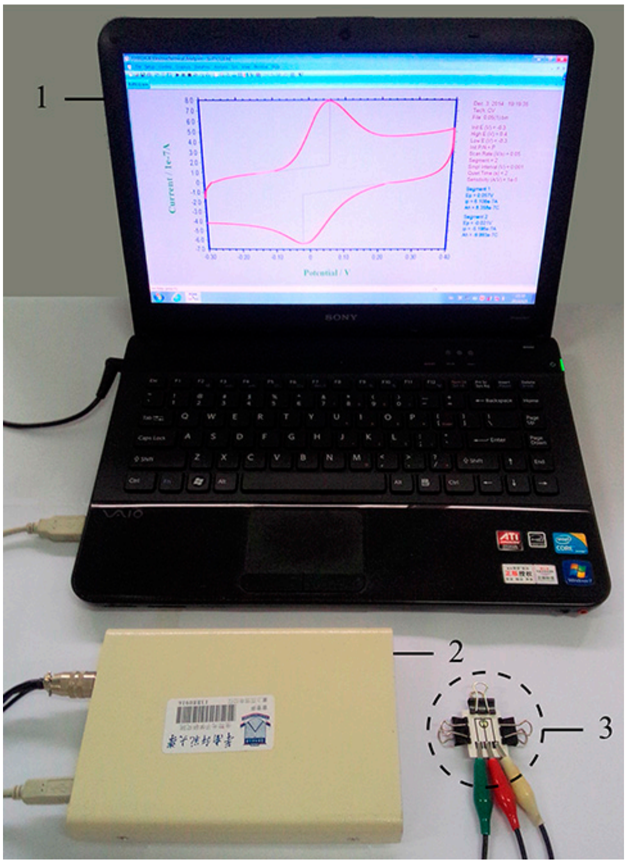

Figure S1. Photograph of the setup. The device principally consists of a personal computer (1), handheld bipotentiostat (2), and droplet-based electrochemical (EC) sensor (3).

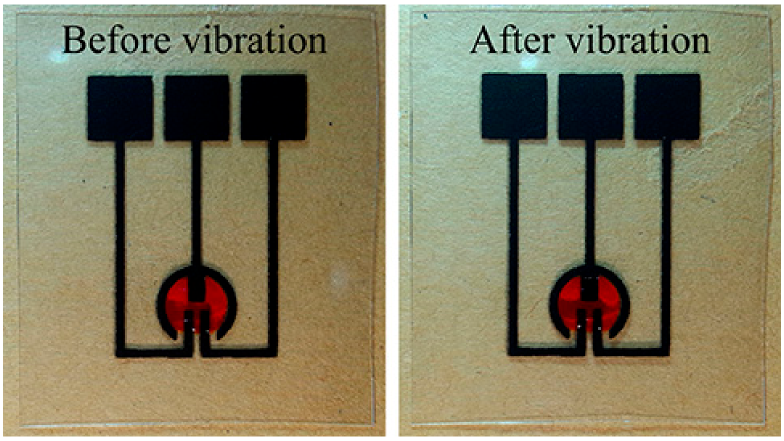

(**A**) (**B**)

Figure S2. Photograph of the droplet-based electrochemical (EC) sensor before (**A**) and after (**B**) vibration. Here, the vibration parameters included as follows: rotational speed (180 rpm), displacement (~2 cm) and duration of perturbation (12 s).

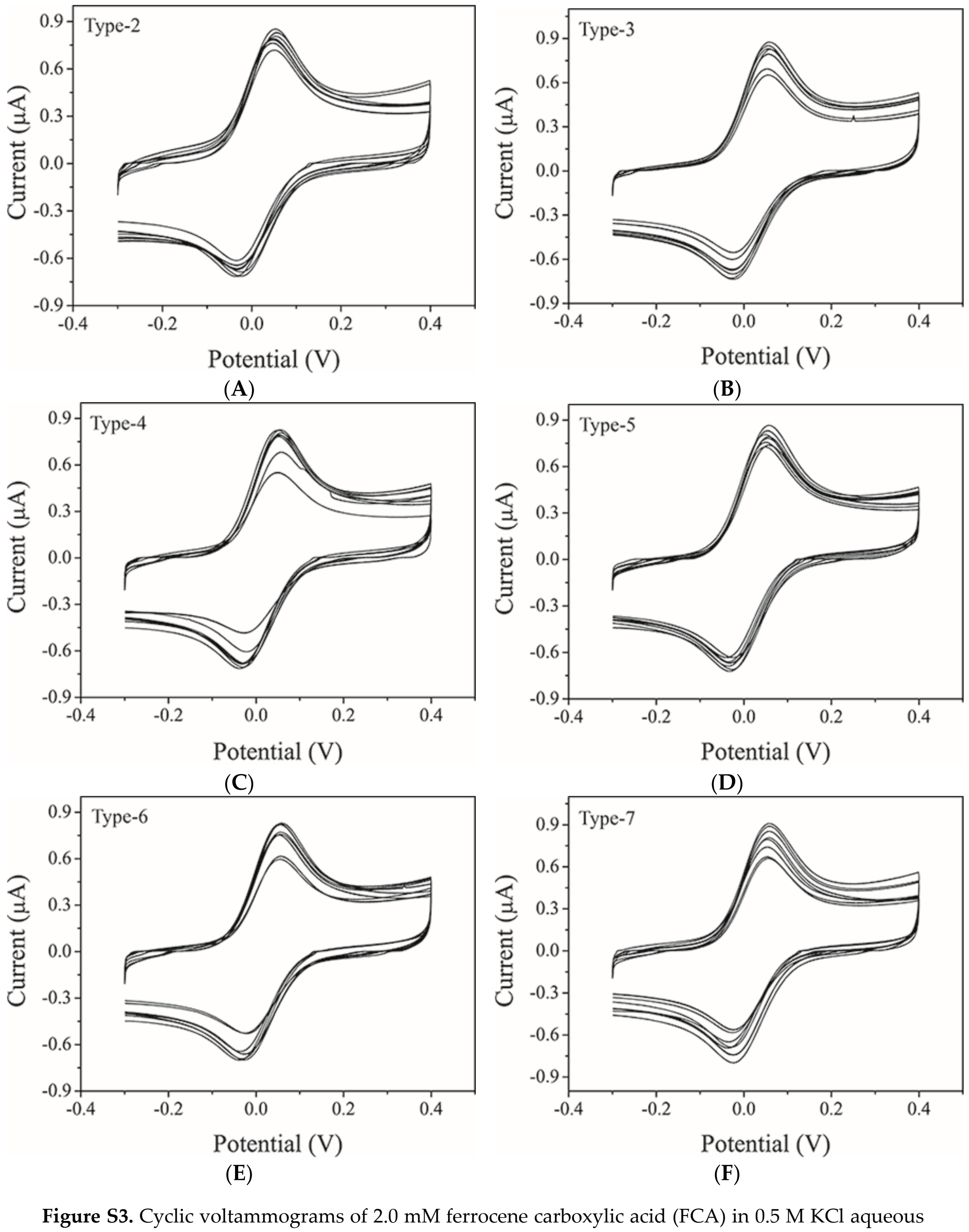

Figure S3. Cyclic voltammograms of 2.0 mM ferrocene carboxylic acid (FCA) in 0.5 M KCl aqueous solution using Type-2 (**A**); Type-3 (**B**); Type-4 (**C**); Type-5 (**D**); Type-6 (**E**) and Type-7 (**F**) electrochemical (EC) sensors (scan rate: 100 mV/s; potential range: −0.3 to 0.4 V versus carbon pseudo-reference electrode). Here, eight independent measurements were performed for each pattern.
